# Advancing the Care of Delirium and Comorbid Dementia

**DOI:** 10.3390/geriatrics7060132

**Published:** 2022-11-23

**Authors:** Alessandro Morandi, Maria Wittmann, Federico Bilotta, Giuseppe Bellelli

**Affiliations:** 1 Azienda Speciale “Cremona Solidale”, 26100 Cremona, Italy; 2 Parc Sanitari Pere Virgili, Vall d’Hebrón Institute of Research, 08023 Barcelona, Spain; 3 Department of Anaesthesiology and Intensive Care Medicine, University Hospital Bonn, 53127 Bonn, Germany; 4 Department Anesthesiology and Critical Care, “Sapienza” University of Rome, 00185 Rome, Italy; 5 School of Medicine and Surgery, University Milano-Bicocca, 20126 Milan, Italy

Delirium is defined as an acute neuropsychiatric disorder characterized by a disturbance in attention and awareness, which develops over a short period of time, with additional disturbances in cognition which are not explained by a pre-existing cognitive impairment [1]. Importantly, the development of delirium is multifactorial and is generally triggered by medical causes, surgery, anesthesia, pain and/or drug administration or withdrawal. Post-operative delirium (POD) usually occurs within the first five days after surgery, and its incidence varies depending on the type of surgery, with the incidence of POD being highest in cardiac surgery, at over 50% [2]. Predisposing risk factors for POD include age, pre-operative cognitive impairment, pre-existing systemic diseases such as heart failure and the number of medications the patient takes daily [3].

When delirium occurs in the context of a pre-existing dementia, it is defined as delirium superimposed on dementia (DSD) [4]. The prevalence of DSD varies according to the studies and to the tools used to diagnose delirium in this population [5,6,7]. The prevalence of dementia is expected to nearly double every 20 years, to 65 million in 2030 and 115 million in 2050. DSD should therefore be considered as a key priority for health care providers. In a large prospective cohort study of older patients admitted to an acute hospital, the prevalence of DSD was about 33% [8]. However, in a recent meta-analysis of 81 studies including 81,536 people with dementia, the pooled DSD prevalence was 48.9% [9]. DSD prevalence was found to be higher in orthopedic (63.2%) and general surgery (62.3%) [9]. The occurrence of delirium in dementia patients is associated with a longer hospital stay, worse functional and cognitive outcomes and higher risk of hospitalization and death than patients without dementia [9]. Several factors are associated with or increase the risk of delirium in this frail population, including hospital-related factors (i.e., delays in surgical procedures in hip fracture patients), illness-related factors (i.e., severity of acute illness, pain, malnutrition, acute infection), medications (i.e., use of psychotropic drugs, polypharmacy), and non-modifiable factors (i.e., age, male gender, severity of dementia and severity of comorbidities) [9].

The development of delirium is indeed multifactorial, and it requires complex and multidisciplinary interventions for the prevention and treatment of DSD [10]. It is indeed essential to systematically use tools for the diagnosis of delirium such as the 4AT, the Confusion Assessment Method or specific tools designed for patients with moderate to severe dementia, such as the 4-DSD [11,12,13]. Other supporting instruments have been proposed, given the difficulties in diagnosing delirium, especially in the advanced stages of dementia. In fact, studies have reported the importance of motor fluctuations for the detection of delirium, given that delirium is not an isolated mental disorder but can affect motor fluctuation as well [14,15,16]. Therefore, in patients with dementia and especially in the advanced stages of dementia, it might be useful to use tools that not only evaluate attention and other cognitive deficits, but also motor performance and changes. It could be hypothesized that we should screen patients for delirium using the modified-Richmond Agitation and Sedation Scale (m-RASS) [17,18], followed by a 4-AT assessment to increase the ability to detect DSD.

There is current evidence that delirium can be prevented using a non-pharmacological multicomponent intervention [10]. The multicomponent intervention adopted includes reorientation, drug reconciliation and the reduction in psychoactive drugs, the promotion of sleep, early mobilization, adequate hydration and nutrition, and the use of vision and hearing devices. An interdisciplinary team involving geriatricians or other medical clinicians, nurses, physiotherapists, occupational therapists, speech therapists, nutritionists, clinical pharmacists and social workers should carry out this multicomponent intervention. There is specific emerging evidence on the role of occupational therapy in the management of patients with delirium and especially in those with delirium and dementia [19,20,21].

In this Special Issue, we aim to provide an overview of delirium in patients with dementia, focusing on the evolution of the epidemiology, providing insights on the current hypothesis of the pathophysiology of DSD and a specific focus on how a delirium diagnosis should be approached in this population. Finally, our focus is to provide clinicians with a practical approach to the management of risk factors for delirium, especially in the context of surgical procedures and postoperative delirium, and elucidate how to implement the multidisciplinary and interdisciplinary management of DSD.
